# Supplementary material for: Polyketide synthase gene clusters in the ericoid mycorrhizal fungus Oidiodendron maius: genome-wide prediction, expression profiling, and functional characterization of knockout mutants
Source: Front Plant Sci. 2026 Sep 16;17:1924430. doi: 10.3389/fpls.2026.1924430 (PMC13623903; doi:10.3389/fpls.2026.1924430)
Supplement: Supplementary file 1 [file SupplementaryFile1.zip › Supplementary Materials/Supplementary Folder 1.zip!/Oidma1_AssemblyScaffolds_Repeatmasked/knownclusterblast/cluster8/jgi.p_Oidma1_108402_mibig_hits.html]

| MIBiG Protein | Description | MIBiG Cluster | MiBiG Product | % ID | % Coverage | BLAST Score | E-value |
| --- | --- | --- | --- | --- | --- | --- | --- |
| CUI25695.1 | monooxygenase,\_FAD-binding\_protein | BGC0001352 | Thiotetronate\_Tu\_3010\_biosynthetic\_gene\_cluster | 36 | 97.2431077694 | 235 | 1.1e-61 |
| ANY57879.1 | PenA | BGC0001372 | Penigequinolone\_biosynthetic\_gene\_cluster | 29 | 106.015037594 | 153 | 4.1e-37 |
| BAG16279.1 | putative\_geranylgeranyl\_diphosphate\_epoxidase | BGC0000632 | Brasilicardin\_A\_biosynthetic\_gene\_cluster | 24 | 90.4761904762 | 59 | 1.4e-08 |
